# The structure of tick-borne encephalitis virus determined at X-ray free-electron lasers. Simulations

**DOI:** 10.1107/S1600577522011341

**Published:** 2023-01-01

**Authors:** Dameli Assalauova, Ivan A. Vartanyants

**Affiliations:** a Deutsches Elektronen-Synchrotron DESY, Notkestrasse 85, 22607 Hamburg, Germany; ESRF and Université Grenoble Alpes, France

**Keywords:** single-particle imaging, XFELs, tick-borne encephalitis virus

## Abstract

The study of virus structures by X-ray free-electron lasers (XFELs) has attracted increased attention in recent decades. Such experiments are based on the collection of 2D diffraction patterns measured at the detector following the application of femtosecond X-ray pulses to biological samples. To prepare an experiment at the European XFEL, the diffraction data for the tick-borne encephalitis virus (TBEV) was simulated with different parameters and the optimal values were identified. Following the necessary steps of a well established data-processing pipeline, the structure of TBEV was obtained. In the structure determination presented, *a priori* knowledge of the simulated virus orientations was used. The efficiency of the proposed pipeline was demonstrated.

## Abstract 2

In this work, a single-particle imaging experiment of tick-borne encephalitis virus at the European XFEL was simulated. These simulations allow the optimal parameters of the experimental setup to be obtained for use in future experiments.

## 1. Introduction

Understanding the structure and functionality of viruses has been an important task in biology, physics, chemistry and medicine over the last centuries. The present COVID-19 pandemic has shown that, without knowledge of the structure and functionality, it is difficult, or even impossible, to develop handling strategies with the SARS-Cov-2 virus. The development of different imaging techniques to study single particles, especially viruses, can bring insight to the structural features of these objects. Nowadays, several methods exist and may be applied. X-ray crystallography is the predominant method for determining the structures of biomolecules with high resolution, but since this requires the crystallization of a protein or virus, its application is not always possible. More often this method is used for single viral proteins or compact, homogeneous, symmetric viral particles (Zhang *et al.*, 2017 ▸; Rossmann, 2014 ▸; Yang & Rao, 2021 ▸). Small viral proteins, especially unstructured ones, are investigated by nuclear magnetic resonance (NMR) (Neira, 2013 ▸). Some membrane proteins are difficult to crystallize due to their hydro­phobicity and the laborious process of manufacturing the quantities of proteins required for crystallization (Choy *et al.*, 2021 ▸). However, knowledge of the structure of these proteins is essential for understanding the functioning of viruses. One way to solve the problems mentioned above is to study the spatial structure of biological particles, using methods such as single-particle imaging (SPI). SPI using cryogenic electron microscopy (cryo-EM) makes it possible to reconstruct the structure of biological particles down to atomic or even interatomic resolution (Choy *et al.*, 2021 ▸; Egelman, 2016 ▸; Callaway, 2020 ▸). The reconstruction is based on many measured projections from different virions. In most cases, this method works well with structurally homogeneous particles (Agard *et al.*, 2014 ▸). However, a frequent case occurs when DNA/RNA is randomly packed in different virions. This leads to blurry electron density in the centre of the particle making the internal structure rather difficult to determine (Lyumkis, 2019 ▸). In addition, when using cryo-EM, the samples must be cooled to liquid-nitro­gen temperature, which makes it difficult to understand the functionality of viruses at room temperature and in their native environment. These limitations can be circumvented using another approach based on the use of X-ray free-electron lasers (XFELs), which are in the early stages of development.

It has been shown that sufficiently intense femtosecond XFEL pulses allow collection of diffraction patterns before radiation damage by Coulomb explosion takes place [so-called ‘diffraction-before-destruction’ approach (Chapman *et al.*, 2006 ▸)]. This gave rise to SPI XFEL experiments (Neutze *et al.*, 2000 ▸; Chapman *et al.*, 2006 ▸; Gaffney & Chapman, 2007 ▸), which were implemented at XFEL facilities built over the last ten years. Currently, these facilities are the most powerful X-ray sources and can produce strong X-ray radiation with the pulse length of several tens of femtoseconds (Emma *et al.*, 2010 ▸; Ishikawa *et al.*, 2012 ▸; Decking *et al.*, 2020 ▸; Kang *et al.*, 2017 ▸). Note that these sources have a high degree of coherence (Vartanyants *et al.*, 2011 ▸; Singer *et al.*, 2012 ▸; Gutt *et al.*, 2012 ▸). It is this important property that makes it possible to use coherent X-ray diffractive imaging (CXDI) methods (Miao *et al.*, 1999 ▸, 2015 ▸) in order to obtain the spatial structure of biological particles.

In spite of a strong push by the community and a whole series of experiments with various biological particles (Seibert *et al.*, 2011 ▸; Ekeberg *et al.*, 2015 ▸; Rose *et al.*, 2018 ▸; Kurta *et al.*, 2017 ▸), progress in SPI experiments has not been as rapid as expected. The resolution in these experiments has been limited to a few tens of nanometres. To better understand and overcome the experimental limitations, the SPI consortium was created several years ago (Aquila *et al.*, 2015 ▸). As a result of the collaboration between the consortium members, a resolution better than 10 nm was obtained for viruses of about 70 nm in size (Rose *et al.*, 2018 ▸; Assalauova *et al.*, 2020 ▸). In a recent SPI experiment at the European XFEL (Hamburg, Germany) with gold nanoparticles, a resolution of 2 nm was achieved (Ayyer *et al.*, 2021 ▸). Thus, the resolution in future SPI experiments with viruses of similar sizes can be expected to be between 2 nm and 10 nm.

During the past decade, significant progress has been made in the development of a data analysis pipeline for SPI experiments to determine the electron density of viruses from the diffraction patterns collected (Gaffney & Chapman, 2007 ▸; Assalauova *et al.*, 2020 ▸). An important step in data analysis – the clustering of 2D diffraction patterns and the selection of diffraction patterns that correspond to the particle under study – can be addressed with different approaches. For example, Assalauova *et al.* (2020 ▸) used a clustering algorithm based on the maximum-likelihood method, which is actively used in cryo-EM (Dempster *et al.*, 1977 ▸). In the work by Assalauova *et al.* (2022 ▸), machine learning methods using a convolutional neural network (CNN) were used for the same purpose. The reconstructed electron density of the virus was obtained by the modal decomposition of several reconstructions of the virus under study (Assalauova *et al.*, 2020 ▸).

For a successful outcome, each SPI experiment requires careful planning. Here, an SPI experiment planned at the European XFEL to investigate the spatial structure of TBEV is discussed. To demonstrate this, an SPI experiment at the European XFEL for two configurations of TBEV was simulated. Various experimental conditions are discussed in detail including the incident photon flux on the sample and the sample-to-detector distance. This work also presents a general data analysis pipeline and the existing structure reconstruction platform (Bobkov *et al.*, 2020 ▸). In this structure determination, *a priori* knowledge of the simulated virus orientations was used and the experimental background signal was neglected.

## 2. Tick-borne encephalitis virus

Tick-borne encephalitis is a viral infectious disease transmitted through tick bites. The endemic area extends from west to east from the Rhine to the Urals and from north to south from Scandinavia to Italy and Greece. Tick-borne encephalitis is usually asymptomatic, but can also cause serious complications, mainly in the form of damage to the nervous system, and can result in disability or even death. There is no cure for tick-borne encephalitis — the main preventive measure is vaccination.

The pathogen of tick-borne encephalitis is a virus belonging to the family *Flaviviridae*, genus *Flavivirus*. In addition to tick-borne encephalitis, flaviviruses cause a number of serious human diseases, including long-known infections such as yellow fever, Dengue fever, West Nile fever, Japanese encephalitis, as well as those newly discovered and capable of rapid spread to new territories such as the Zika fever (Barrows *et al.*, 2018 ▸). Several million cases of flavivirus infections are reported worldwide each year (Barrows *et al.*, 2018 ▸; Pierson & Diamond, 2020 ▸).

All viruses of this family are enveloped viruses with a virion diameter of ∼50 nm. The virion core consists of a single-stranded (+)RNA molecule surrounded by protein C. It is covered on top by a lipid membrane, in which two proteins are embedded: the membrane protein M and the virion surface protein E. Glycoprotein E is mainly responsible for the first stages of viral infection and is the target of most neutralizing antibodies (Pierson & Diamond, 2020 ▸). The structure of these viruses accounts for their natural heterogeneity: mature, immature, semi-mature and so-called ‘broken’, *i.e.* deformed particles that are formed in the samples during maturation (Füzik *et al.*, 2018 ▸; Pichkur *et al.*, 2020 ▸). This makes it difficult to obtain the structures of flavivirus virions by X-ray crystallography, since heterogeneity prevents obtaining ordered crystals. In this regard, the method for obtaining flavivirus virion structures is cryo-EM. Currently, the Protein Data Bank (PDB; https://www.rcsb.org/) contains more than 40 structures of various flaviviruses.

**Figure 1:**
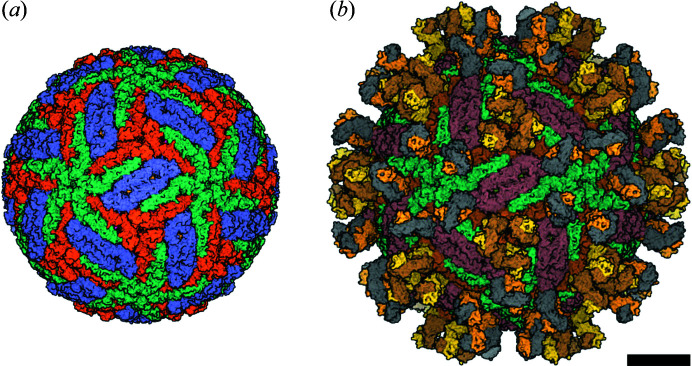
Cryo-EM structure of TBEV. (*a*) Structure of the mature TBEV particle 5o6a. (*b*) Structure of the complex with the Fab-fragment of the neutralizing monoclonal antibody 5o6v. The structures of TBEV were taken from the PDB (https://www.rcsb.org/). The scale bar is 10 nm.

The cryo-EM method requires careful sample preparation (Pichkur *et al.*, 2020 ▸) and the maintenance of a fairly high concentration of homogeneous particles of the same type, usually mature or immature virions, which are the most symmetrical. Viral particles with antigen-binding fragments (Fab-fragments) that neutralize antibodies (Long *et al.*, 2019 ▸; Rey *et al.*, 2018 ▸) are also studied. Two TBEV structures have been obtained by cryo-EM with 3.9 Å resolution (Füzik *et al.*, 2018 ▸): the structure of the mature virion complex [PDB entry 5o6a; see Fig. 1 ▸(*a*)] and the structure of the mature virion complex with the Fab-fragment of the mouse monoclonal antibody 19/1786 [PDB entry 5o6v, see Fig. 1 ▸(*b*)]. We used both structures of the virus as the model object when simulating diffraction data.

## 3. Data simulations for the SPI experiment on the European XFEL with TBEV

**Figure 2:**
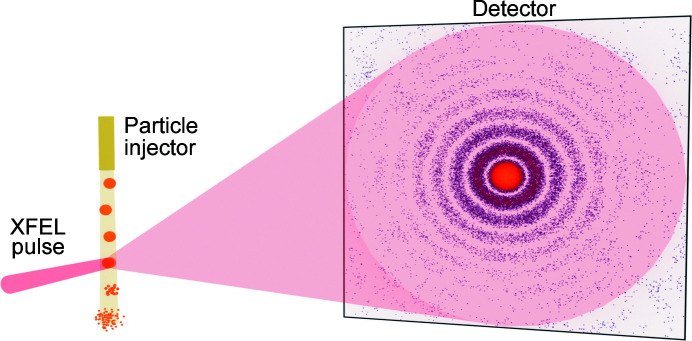
Experimental setup of an SPI-experiment to determine the spatial structure of a single biological particle. The specimen of the particle is injected into an X-ray laser beam in a random orientation, and the scattered radiation is recorded by the detector.

One of the goals of the TBEV simulations was to plan a future SPI experiment at the European XFEL at the Single Particles, Clusters and Biomolecules (SPB) beamline. A typical experimental setup of the SPI experiment is well known and described in detail by Gaffney & Chapman (2007 ▸). In such an experiment, femtosecond X-ray pulses are focused onto single virus specimens in random orientations (see Fig. 2 ▸). The particles are destroyed following the interaction with the X-ray beam, but 2D patterns containing diffraction of the initial virus state (Gorobtsov *et al.*, 2015 ▸) are recorded on the detector. Reconstructing the spatial structure of a virus from these 2D diffraction patterns is the main task of SPI.

The success of complex SPI experiments depends on many different parameters. Parameters that can be evaluated in advance by simulation include the incident photon flux and the sample-to-detector distance. The scattered signal clearly depends on the intensity of the incident X-ray beam. At the European XFEL its intensity is 1–4 mJ per pulse, which corresponds to 10^11^–10^12^ photons per pulse. Since in the planned experiment the size of the focal spot of the X-ray beam is 300 nm, it is natural to assume that there will be no more than 10^12^ photons per pulse in the focal spot.

The lower the energy of the incident photons, the stronger the scattered radiation. Therefore, the planned photon energy is 6 keV (wavelength 2.07 Å), as this energy is the lowest possible energy that can be used at the SPB station. The European XFEL SPB beamline uses an AGIPD 1 Mpx detector (Allahgholi *et al.*, 2019 ▸) with a size of 1024 × 1024 pixels (one pixel is 200 µm × 200 µm). The parameters of the SPB beamline described above were used in the simulation of diffraction patterns using the *MOLTRANS* program developed at DESY.

**Figure 3:**
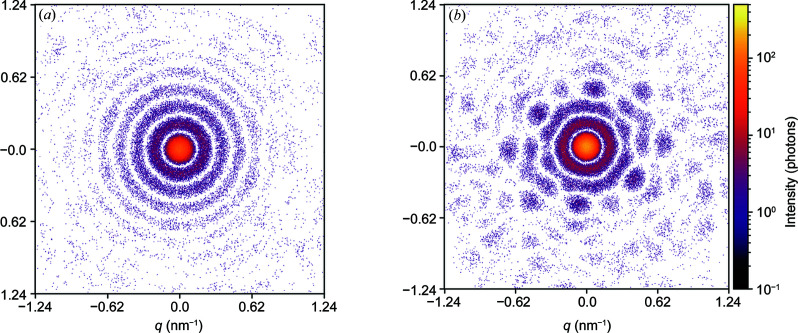
Diffraction patterns from single objects in a random orientation: (*a*) 5o6a [for TBEV in Fig. 1 ▸(*a*)]; (*b*) 5o6v [for TBEV with Fab-fragments in Fig. 1 ▸(*b*)]. Simulation parameters: 2.07 Å wavelength, 300 nm X-ray beam focus, 512 × 512 pixel detector size, 400 µm × 400 µm pixel size, 2.5 m sample-to-detector distance and 10^12^ photons signal intensity at the focus.

First of all, it is of interest to compare the diffraction patterns of the two available virus types: 5o6a and 5o6v. The global symmetry of both is icosahedral, but the 5o6a structure has a pronounced spherical shape, and the diffraction pattern from such an object consists of concentric rings. The structure of 5o6v looks different to that of 5o6a in reciprocal space. Due to the antigen-binding fragments, sixth-order symmetry can be observed in the diffraction patterns, indicating the appearance of characteristic features of the structure. Examples of diffraction patterns from two structures for a random orientation are shown in Fig. 3 ▸.

For the demonstration and optimization of experimental parameters, we used the 5o6v structure [see Fig. 1 ▸(*b*)]. The scattered signal recorded by the detector depends on the parameters of the experimental setup as well as on the sample characteristics, *i.e.* the larger the object, the higher the intensity of the scattered signal. Note that the cryo-EM structures taken from the PDB bank and used in the present work describe the surface protein E and membrane protein M and do not characterize the inner nucleocapsid formed by protein C and the RNA structure chain. Naturally, in the SPI experiment, the presence of the RNA nucleocapsid in the particles will contribute to the scattered signal on the detector.

**Figure 4:**
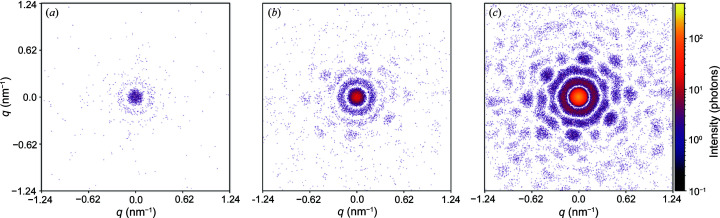
Diffraction patterns from a single TBEV in a random orientation. Signal intensities: (*a*) 10^10^ photons, (*b*) 10^11^ photons and (*c*) 10^12^ photons at the focus. The simulated sample-to-detector distance is 2.5 m. Detector parameters are the same as in Fig. 3 ▸.

Fig. 4 ▸ shows three diffraction patterns from a single virus with different photon flux in the focus of the X-ray beam. This figure demonstrates that the diffraction patterns contain more structural features of the particle with increasing photon flux from 10^10^–10^12^ photons at the X-ray beam focus. If the scattering signal from the virus is too weak [see Fig. 4 ▸(*a*)], the features of the object are less visible, making further data analysis steps difficult. The minimum number of recorded photons per diffraction pattern needed for a successful reconstruction has been actively studied (Ayyer *et al.*, 2015 ▸, 2019 ▸; Giewekemeyer *et al.*, 2019 ▸; Ekeberg *et al.*, 2022 ▸). It was shown (Poudyal *et al.*, 2020 ▸) that low intensity within measured diffraction patterns can be compensated with an increased number of diffraction patterns collected during the experiment. With the parameters used to obtain the diffraction pattern in Fig. 4 ▸(*c*), the scattered signal is determined to a value of 1.24 nm^−1^ in reciprocal space, which corresponds to a resolution of 5 nm in real space. From the simulations (see Fig. 4 ▸) we can conclude that the maximum signal intensity of 10^11^–10^12^ photons at the X-ray beam focus, which is achievable at the European XFEL, is necessary for the experiment to succeed.

**Figure 5:**
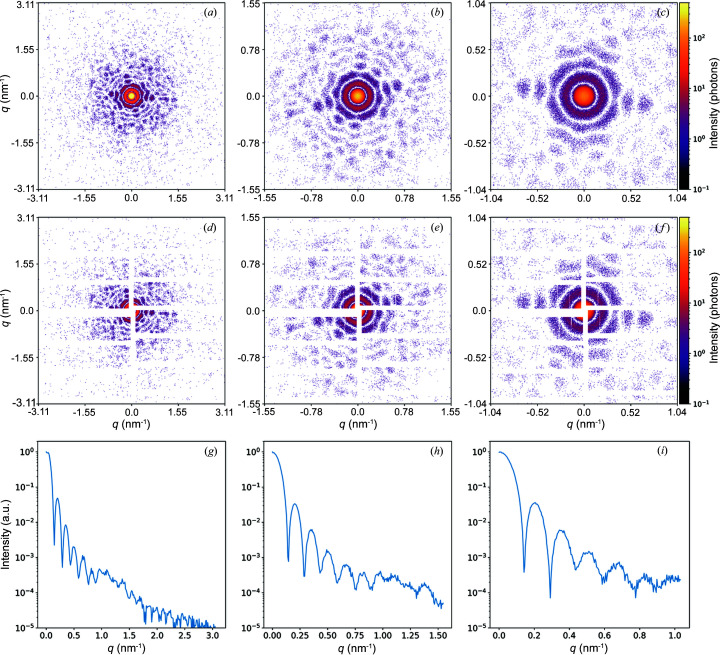
Diffraction patterns from a single TBEV in a random orientation. At sample-to-detector distances of (*a*, *d*, *g*) 1 m; (*b*, *e*, *h*) 2 m; and (*c*, *f*, *i*) 3 m. (*d*)–(*f*) Examples of diffraction patterns with the detector mask superimposed on them. (*g*)–(*i*) Angular averaged intensities of the diffraction patterns shown in (*a*)–(*c*). Detector parameters are the same as in Fig. 3 ▸.

Another important parameter of the SPI experiment that can be analysed through simulation is the sample-to-detector distance. If the distance is too small, it will not provide pronounced diffraction from the sample, but it will allow for a high resolution to be obtained. If the distance is too large, however, the desired resolution will not be achievable. Examples of diffraction patterns with different sample-to-detector distances from 1 m to 3 m are shown in Figs. 5 ▸(*a*)–5(*c*). An angle-averaged intensity was plotted for each case and is shown in Figs. 5 ▸(*g*)–5(*i*). As expected, at the shortest distance of 1 m the diffraction pattern shows the characteristic features of the virus structure and the resolution in real space reaches 2 nm. As the sample-to-detector distance is increased to 2 m, these features become more pronounced, but the resolution in real space drops to 4 nm. At the maximum distance simulated of 3 m, the diffraction pattern from the virus is clearly distinguishable; in Fig. 5 ▸(*i*) the characteristic rings are clearly visible. But the resolution in reciprocal space is limited to 6.3 nm.

An important factor when choosing the optimal sample-to-detector distance is the structure of the detector panels. Their configuration has a high dependence on the sample and experimental setup. In practice, the detector panels are not placed together; there is a distance between them. There is also a gap in the centre of the detector for the direct (central) beam to pass through. Simulated diffraction patterns emulating an experiment with the superimposed geometry of gaps between the detector panels are shown in Figs. 5 ▸(*d*)–5(*f*). The detector geometry used is representative of SPI experiments at the SPB beamline of the European XFEL. Fig. 5 ▸(*d*) shows that a large part of the central diffraction peak at a distance of 1 m is not determined because of the panel positions in the central part of the detector. Information about the size of the central peak must be recorded and is essential when reconstructing the object. It is also important to take this into account when planning the experiment, in particular, when choosing the optimal sample-to-detector distance. From the analysis of the simulations performed (see Fig. 5 ▸) with different sample-to-detector distances, we can conclude that a distance of 2–3 m is preferable. In this case, the detector geometry makes it possible to distinguish all structural features of the virus, and the momentum transfer vector *q* reaches a value of 1.04–1.55 nm^−1^ in reciprocal space, which corresponds to a resolution of 4–6 nm in real space.

## 4. Data analysis pipeline of SPI experiment to obtain the spatial structure of the object

The data analysis pipeline of SPI experiments aims to obtain the 3D structure of the object from the measured diffraction patterns collected in the experiment. The structure of the object is determined by the electron density distribution, and the measured 2D diffraction patterns contain information about the reciprocal space, which is a 3D Fourier transform image of the electron density. By determining the orientation of the series of 2D diffraction patterns, they can be assembled into one 3D volume in reciprocal space. The corresponding scattered phase values of this 3D volume then need to be recovered, allowing the electron density distribution to be obtained through the inverse Fourier transform.

To account for the specifics of the experiment, additional steps are included in the data analysis procedure (Rose *et al.*, 2018 ▸; Bobkov *et al.*, 2020 ▸; Assalauova *et al.*, 2020 ▸). First, due to the fact that only a small fraction of the images contain diffraction patterns, empty images are filtered out from the analysis. Second, before starting the analysis of the diffraction patterns, the position of the centre of the diffraction pattern relative to the detector position should be precisely determined. Then, among all diffraction patterns, those which contain scattering signal from only a single virus are selected. For this purpose, first the size of the samples is estimated from the diffraction patterns and those with a reasonable size distribution are selected. Then, the selected patterns are classified according to the features of the diffraction patterns, which are related to the features of the sample structure in real space. Thus, only the patterns related to the object studied are distinguished, *i.e.* so-called single hits. From our experience, a significant number of diffraction patterns correspond to impurities or water droplets, or contain diffraction from several objects combined. Therefore, the quality of diffraction pattern classification directly influences the structure resolution obtained. If the quality of single-hit classification is insufficient, structure reconstruction becomes impossible.

The combination of diffraction patterns in 3D reciprocal space requires determination of the relative orientation of the diffraction patterns relative to each other. This is directly related to the orientations of the object inserted in the X-ray pulse during the experiment. The orientation determination and the reconstruction of the reciprocal space volume are based on the maximum-likelihood method (Loh & Elser, 2009 ▸) implemented in the expand–maximize–compress (EMC) algorithm (Ayyer *et al.*, 2016 ▸). It was shown to work well and to orient relatively small amounts of diffraction data with missing areas and a small number of photons. Once the reciprocal space volume has been reconstructed, the background signal, usually caused by parasitic scattering on the elements of the experimental setup, is corrected. As the background signal does not depend on the orientation of the sample, the background correction is performed on the reconstructed 3D intensity volume in reciprocal space. At this stage the influence of the background is averaged, making it easier to correct.

Next, the phases of the diffraction patterns are recovered in reciprocal space and the real space structure is determined. For this purpose, iterative phase retrieval algorithms are used (Fienup, 1982 ▸, 2013 ▸; Marchesini *et al.*, 2003 ▸). Finally, the resolution of the resulting electron density distribution is evaluated.

The pipeline presented was tested with data from several SPI experiments obtained at LCLS (Stanford, USA) and the European XFEL (Sobolev *et al.*, 2020 ▸). Processing the experimental SPI data according to the pipeline allowed for a significant improvement in the structure recovery (Rose *et al.*, 2018 ▸; Assalauova *et al.*, 2020 ▸). Software was developed to implement all the stages of the pipeline, and a platform for automated data processing of SPI XFEL experiments was created (Bobkov *et al.*, 2020 ▸). The platform includes containerized software that is integrated into a software pipeline, which provides automatic processing from experimental data to structure reconstruction. The platform can be quickly run on any computing architecture with container support [*e.g.* Docker (Merkel, 2014 ▸)], as well as in computing clusters run by Kubernetes (https://kubernetes.io/). Information about the developed software (Bobkov *et al.*, 2020 ▸) and the automatic data-processing platform is publicly available (https://gitlab.com/spi_xfel).

## 5. Spatial structure of the TBEV from simulated data

In this work, we adapted the above-mentioned data analysis pipeline for the simulated diffraction patterns from TBEV both with and without Fab-fragments, with the goal to obtain information on the respective spatial structures. Considering the analysis performed in Section 3, 1000 diffraction patterns [without the superimposed detector mask, as shown in Figs. 5 ▸(*a*)–5(*c*)] were created for each TBEV type (see Fig. 1 ▸). The detector was simulated with a 128 × 128 pixel size, and with a 1.6 mm × 1.6 mm pixel size which represents an eight times binned configuration. These dimensions were set in order to match the real size of the AGIPD 1 Mpx detector while also saving on computational time. The simulated sample-to-detector distance was 2.1 m, and the signal intensity was 10^11^ photons at the focus for TBEV in random orientations. The sample-to-detector distance was chosen according to the geometry of the planned experiment at the SPB beamline of the European XFEL. The number of simulated diffraction patterns (1000) was based on the existing research (Poudyal *et al.*, 2020 ▸) showing the dependence between this number, the experimental parameters and a spatial resolution of several nanometres or less. The number of simulated diffraction patterns can be also estimated from the experimental data (Rose *et al.*, 2018 ▸; Assalauova *et al.*, 2020 ▸, 2022 ▸). This number depends on the studied particle and experimental conditions.

**Figure 6:**
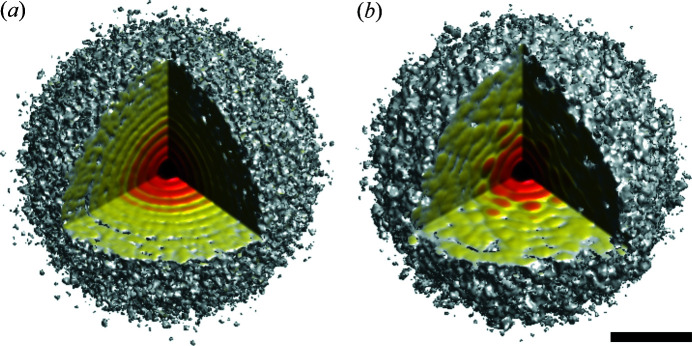
Diffraction 3D intensity volume in reciprocal space: (*a*) 5o6a [for TBEV in Fig. 1 ▸(*a*)]; (*b*) 5o6v [for TBEV with Fab-fragments, Fig. 1 ▸(*b*)]. The scale bar is 1 nm^−1^.

Since only the structure of the TBEV obtained by cryo-EM (Füzik *et al.*, 2018 ▸) was used in the simulation, clustering and classification of the diffraction patterns by object type was not required. As the orientation of the particle in the simulations is known, combining the data into a 3D diffraction intensity volume in reciprocal space was done according to the known orientations of the virus. The result of the 3D volume in reciprocal space is shown in Fig. 6 ▸. For virus 5o6a [see Fig. 6 ▸(*a*)], as expected, concentric rings are observed. Due to the presence of Fab-fragments in the structure of virus 5o6v, diffraction fringes in reciprocal space are observed [see Fig. 6 ▸(*b*)].

As the experimental background signal strongly depends on the experiment and setup, it was not included in the diffraction patterns, and therefore no correction step was required. The next step is to reconstruct the scattering phases and structure of the object. As described earlier, iterative phase retrieval algorithms are used for this task (Fienup, 1982 ▸, 2013 ▸; Marchesini *et al.*, 2003 ▸). These algorithms are based on the Fourier transform between real and reciprocal spaces using two constraints: in reciprocal space, the signal amplitude is set to be equal to the experimentally measured values, and, in real space, the object occupies a limited volume with an approximate size that is known in advance.

**Figure 7:**
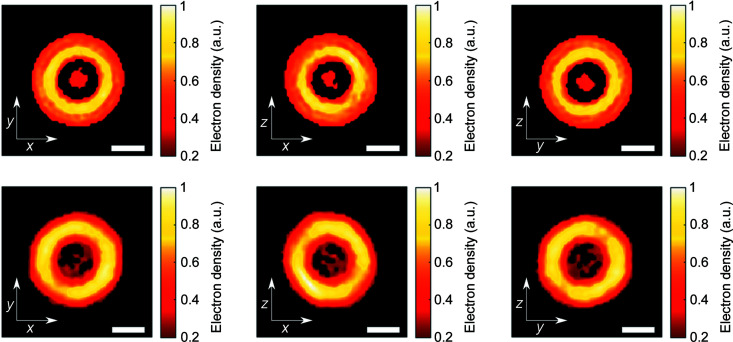
Central slices of the TBEV reconstructions. Upper row: 5o6a; lower row: 5o6v. Electron density values are normalized to the maximum, values less than 0.2 are shown in black. The scale bar is 20 nm.

To obtain the spatial structure of the virus (for 5o6a and 5o6v) the following combination of algorithms was used: 100 iterations of continuous hybrid input–output, followed by 200 iterations of error reduction with an alternating shrink-wrap algorithm every 10 iterations with a threshold value of 0.2. This combination of algorithms was repeated four times for one reconstruction, resulting in a total of 1200 iterations. This process was performed for 30 reconstructions which were then averaged using the mode decomposition method described by Assalauova *et al.* (2020 ▸). The main mode from the decomposition was considered as the final spatial structure, and the results for both TBEV structures are shown in Fig. 7 ▸.

The spatial structures of 5o6a and 5o6v have a ring shape with no density inside the particle, as in the data used for the simulation of diffraction patterns. From the result we can see that, due to the low resolution, Fab-fragments in the structure of 5o6v cannot be distinguished. Thus, the sizes of both structures are about 60 nm. This corresponds to the size of the 5o6v structure (∼57 nm) obtained with cryo-EM (Füzik *et al.*, 2018 ▸). At the same time, the size of the virus obtained by the reconstruction is larger than the size of 5o6a [∼47 nm (Füzik *et al.*, 2018 ▸)]. Note that the structures 5o6a and 5o6v used for the simulations did not contain electron density inside (internal RNA).

## 6. Summary and outlook

An analysis of simulated diffraction patterns for an SPI experiment is presented. SPI allows the spatial structure of biological nanoparticles to be obtained using intense XFEL femtosecond pulses. Such experiments require careful preparation and planning. To prepare the experimental setup and efficiently use the beam time, some parameters can be estimated in advance, for example, by means of simulation. We have presented an analysis of simulated diffraction patterns for an SPI experiment on TBEV.

TBEV was chosen as the studied object, for which the structure of the outer envelope was known from cryo-EM (Füzik *et al.*, 2018 ▸). The size and relative homogeneity of TBEV make it a good object of study in SPI experiments at XFELs.

Two TBEV structures were used to simulate diffraction patterns: a mature virion complex (PDB entry 5o6a) and a mature virion with a Fab-fragment (PDB entry 5o6v). These two structures produce different diffraction patterns in reciprocal space. In the case of 5o6a, only concentric rings were distinguishable in diffraction patterns, whereas, for 5o6v, one can observe characteristic features of the structure associated with ‘spikes’ of Fab-fragments on the surface of the viral particle.

In order to prepare the SPI experiment at the European XFEL, the following parameters were varied during diffraction pattern simulations: the X-ray beam intensity at the focus and the sample-to-detector distance. With the help of simulations, it was possible to determine the optimal parameters and use them in the preparation of the experiment. For the SPB beamline of the European XFEL the following parameters were identified as optimal: 10^11^–10^12^ photon signal intensity at the focus and 2–3 m sample-to-detector distance.

Only the necessary steps of the SPI data analysis pipeline were used for the simulated data. These were the merging of the diffraction data into a 3D volume and the reconstruction of the scattering phases and object structure. Using iterative phase retrieval algorithms, 30 individual reconstructions of each virus were obtained and subsequently averaged by a modal decomposition method. The final structures for 5o6a and 5o6v were then chosen as the main modes from the decomposition. The results of the analysis are as follows. First, for the 5o6v structure it is impossible to distinguish Fab-fragments due to the low resolution. Second, for both structures (5o6a and 5o6v) a ring corresponding to the virus membrane was present with no density inside. This is expected as it matches the original structure used for simulations.

In this study, several steps of the SPI data analysis pipeline were not used, such as background correction, single hit diffraction pattern classification and orientation determination. In real SPI experiments, these steps cannot be avoided and have to be performed very carefully to obtain the final particle structure with high resolution. Each of the steps mentioned above have become the study of separate research: single hit classification (Bobkov *et al.*, 2015 ▸; Shi *et al.*, 2019 ▸; Cruz-Chú *et al.*, 2021 ▸; Ignatenko *et al.*, 2021 ▸; Assalauova *et al.*, 2022 ▸), orientation determination (Loh & Elser, 2009 ▸; Ayyer *et al.*, 2016 ▸) and background subtraction (Rose *et al.*, 2018 ▸; Kurta *et al.*, 2017 ▸; Lundholm *et al.*, 2018 ▸).

The present study using simulated data shows the potential for SPI experiments whose ultimate goal is high-resolution imaging. Although electron ptychography SPI can now achieve a 0.2 Å resolution (Chen *et al.*, 2020 ▸), SPI at XFEL facilities has not yet reached atomic resolution. A higher number of photons scattered on the sample in SPI can increase the diffracted signal; however, this approach is limited and the signal cannot be raised infinitely (Gorobtsov *et al.*, 2015 ▸). One of the factors is the number of collected diffraction patterns during the experiment. Megahertz facilities, such as the European XFEL (Decking *et al.*, 2020 ▸; Mancuso *et al.*, 2019 ▸), allow the necessary amount of data to be obtained in a shorter time. The first experiments have already demonstrated the feasibility of megahertz SPI data acquisition (Sobolev *et al.*, 2020 ▸), which plays a great role for future progress in SPI experiments performed at XFELs. A sufficient number of diffraction patterns with an appropriate photon signal recorded by special detectors, effective sample delivery system and novel data analysis techniques for single-particle classification and orientation determination can open new possibilities of SPI with XFELs to reach its goal of high-resolution imaging of biological particles.

## References

[bb1] Agard, D., Cheng, Y., Glaeser, R. M. & Subramaniam, S. (2014). *Adv. Imaging Electron. Phys.* **185**, 113–137.

[bb2] Allahgholi, A., Becker, J., Delfs, A., Dinapoli, R., Goettlicher, P., Greiffenberg, D., Henrich, B., Hirsemann, H., Kuhn, M., Klanner, R., Klyuev, A., Krueger, H., Lange, S., Laurus, T., Marras, A., Mezza, D., Mozzanica, A., Niemann, M., Poehlsen, J., Schwandt, J., Sheviakov, I., Shi, X., Smoljanin, S., Steffen, L., Sztuk-Dambietz, J., Trunk, U., Xia, Q., Zeribi, M., Zhang, J., Zimmer, M., Schmitt, B. & Graafsma, H. (2019). *J. Synchrotron Rad.* **26**, 74–82. 10.1107/S1600577518016077 PMC6337892 30655470

[bb3] Aquila, A., Barty, A., Bostedt, C., Boutet, S., Carini, G., dePonte, D., Drell, P., Doniach, S., Downing, K. H., Earnest, T., Elmlund, H., Elser, V., Gühr, M., Hajdu, J., Hastings, J., Hau-Riege, S. P., Huang, Z., Lattman, E. E., Maia, F. R. N. C., Marchesini, S., Ourmazd, A., Pellegrini, C., Santra, R., Schlichting, I., Schroer, C., Spence, J. C. H., Vartanyants, I. A., Wakatsuki, S., Weis, W. I. & Williams, G. J. (2015). *Struct. Dyn.* **2**, 041701. 10.1063/1.4918726 PMC4711616 26798801

[bb4] Assalauova, D., Ignatenko, A., Isensee, F., Trofimova, D. & Vartanyants, I. A. (2022). *J. Appl. Cryst.* **55**, 444–454. 10.1107/S1600576722002667 PMC9172041 35719305

[bb5] Assalauova, D., Kim, Y. Y., Bobkov, S., Khubbutdinov, R., Rose, M., Alvarez, R., Andreasson, J., Balaur, E., Contreras, A., DeMirci, H., Gelisio, L., Hajdu, J., Hunter, M. S., Kurta, R. P., Li, H., McFadden, M., Nazari, R., Schwander, P., Teslyuk, A., Walter, P., Xavier, P. L., Yoon, C. H., Zaare, S., Ilyin, V. A., Kirian, R. A., Hogue, B. G., Aquila, A. & Vartanyants, I. A. (2020). *IUCrJ*, **7**, 1102–1113.

[bb6] Ayyer, K., Geloni, G., Kocharyan, V., Saldin, E., Serkez, S., Yefanov, O. & Zagorodnov, I. (2015). *Struct. Dyn.* **2**, 041702. 10.1063/1.4919301 PMC4711618 26798802

[bb7] Ayyer, K., Lan, T.-Y., Elser, V. & Loh, N. D. (2016). *J. Appl. Cryst.* **49**, 1320–1335. 10.1107/S1600576716008165 PMC4970497 27504078

[bb8] Ayyer, K., Morgan, A. J., Aquila, A., DeMirci, H., Hogue, B. G., Kirian, R. A., Xavier, P. L., Yoon, C. H., Chapman, H. N. & Barty, A. (2019). *Opt. Express*, **27**, 37816. 10.1364/OE.27.037816 31878556

[bb9] Ayyer, K., Xavier, P. L., Bielecki, J., Shen, Z., Daurer, B. J., Samanta, A. K., Awel, S., Bean, R., Barty, A., Bergemann, M., Ekeberg, T., Estillore, A. D., Fangohr, H., Giewekemeyer, K., Hunter, M. S., Karnevskiy, M., Kirian, R. A., Kirkwood, H., Kim, Y., Koliyadu, J., Lange, H., Letrun, R., Lübke, J., Michelat, T., Morgan, A. J., Roth, N., Sato, T., Sikorski, M., Schulz, F., Spence, J. C. H., Vagovic, P., Wollweber, T., Worbs, L., Yefanov, O., Zhuang, Y., Maia, F. R. N. C., Horke, D. A., Küpper, J., Loh, N. D., Mancuso, A. P. & Chapman, H. N. (2021). *Optica*, **8**, 15.

[bb10] Barrows, N. J., Campos, R. K., Liao, K.-C., Prasanth, K. R., Soto-Acosta, R., Yeh, S.-C., Schott-Lerner, G., Pompon, J., Sessions, O. M., Bradrick, S. S. & Garcia-Blanco, M. A. (2018). *Chem. Rev.* **118**, 4448–4482. 10.1021/acs.chemrev.7b00719 PMC5937540 29652486

[bb11] Bobkov, S. A., Teslyuk, A. B., Baymukhametov, T. N., Pichkur, E. B., Chesnokov, Y. M., Assalauova, D., Poyda, A. A., Novikov, A. M., Zolotarev, S. I., Ikonnikova, K. A., Velikhov, V. E., Vartanyants, I. A., Vasiliev, A. L. & Ilyin, V. A. (2020). *Crystallogr. Rep.* **65**, 1081–1092.

[bb12] Bobkov, S. A., Teslyuk, A. B., Kurta, R. P., Gorobtsov, O. Yu., Yefanov, O. M., Ilyin, V. A., Senin, R. A. & Vartanyants, I. A. (2015). *J. Synchrotron Rad.* **22**, 1345–1352. 10.1107/S1600577515017348 26524297

[bb13] Callaway, E. (2020). *Nature*, **578**, 201. 10.1038/d41586-020-00341-9 32047310

[bb14] Chapman, H. N., Barty, A., Bogan, M. J., Boutet, S., Frank, M., Hau-Riege, S. P., Marchesini, S., Woods, B. W., Bajt, S., Benner, W. H., London, R. A., Plönjes, E., Kuhlmann, M., Treusch, R., Düsterer, S., Tschentscher, T., Schneider, J. R., Spiller, E., Möller, T., Bostedt, C., Hoener, M., Shapiro, D. A., Hodgson, K. O., van der Spoel, D., Burmeister, F., Bergh, M., Caleman, C., Huldt, G., Seibert, M. M., Maia, F. R. N. C., Lee, R. W., Szöke, A., Timneanu, N. & Hajdu, J. (2006). *Nat. Phys.* **2**, 839–843.

[bb15] Chen, Z., Odstrcil, M., Jiang, Y., Han, Y., Chiu, M.-H., Li, L.-J. & Muller, D. A. (2020). *Nat. Commun.* **11**, 2994. 10.1038/s41467-020-16688-6 PMC7293311 32533001

[bb16] Choy, B. C., Cater, R. J., Mancia, F. & Pryor, E. E. (2021). *Biochim. Biophys. Acta*, **1863**, 183533. 10.1016/j.bbamem.2020.183533 PMC7856071 33340490

[bb17] Cruz-Chú, E. R., Hosseinizadeh, A., Mashayekhi, G., Fung, R., Ourmazd, A. & Schwander, P. (2021). *Struct. Dyn.* **8**, 014701. 10.1063/4.0000060 PMC7902084 33644252

[bb18] Decking, W., Abeghyan, S., Abramian, P., Abramsky, A., Aguirre, A., Albrecht, C., Alou, P., Altarelli, M., Altmann, P., Amyan, K., Anashin, V., Apostolov, E., Appel, K., Auguste, D., Ayvazyan, V., Baark, S., Babies, F., Baboi, N., Bak, P., Balandin, V., Baldinger, R., Baranasic, B., Barbanotti, S., Belikov, O., Belokurov, V., Belova, L., Belyakov, V., Berry, S., Bertucci, M., Beutner, B., Block, A., Blöcher, M., Böckmann, T., Bohm, C., Böhnert, M., Bondar, V., Bondarchuk, E., Bonezzi, M., Borowiec, P., Bösch, C., Bösenberg, U., Bosotti, A., Böspflug, R., Bousonville, M., Boyd, E., Bozhko, Y., Brand, A., Branlard, J., Briechle, S., Brinker, F., Brinker, S., Brinkmann, R., Brockhauser, S., Brovko, O., Brück, H., Brüdgam, A., Butkowski, L., Büttner, T., Calero, J., Castro-Carballo, E., Cattalanotto, G., Charrier, J., Chen, J., Cherepenko, A., Cheskidov, V., Chiodini, M., Chong, A., Choroba, S., Chorowski, M., Churanov, D., Cichalewski, W., Clausen, M., Clement, W., Cloué, C., Cobos, J. A., Coppola, N., Cunis, S., Czuba, K., Czwalinna, M., D’Almagne, B., Dammann, J., Danared, H., de Zubiaurre Wagner, A., Delfs, A., Delfs, T., Dietrich, F., Dietrich, T., Dohlus, M., Dommach, M., Donat, A., Dong, X., Doynikov, N., Dressel, M., Duda, M., Duda, P., Eckoldt, H., Ehsan, W., Eidam, J., Eints, F., Engling, C., Englisch, U., Ermakov, A., Escherich, K., Eschke, J., Saldin, E., Faesing, M., Fallou, A., Felber, M., Fenner, M., Fernandes, B., Fernández, J. M., Feuker, S., Filippakopoulos, K., Floettmann, K., Fogel, V., Fontaine, M., Francés, A., Martin, I. F., Freund, W., Freyermuth, T., Friedland, M., Fröhlich, L., Fusetti, M., Fydrych, J., Gallas, A., García, O., Garcia-Tabares, L., Geloni, G., Gerasimova, N., Gerth, C., Geßler, P., Gharibyan, V., Gloor, M., Głowinkowski, J., Goessel, A., Gołębiewski, Z., Golubeva, N., Grabowski, W., Graeff, W., Grebentsov, A., Grecki, M., Grevsmuehl, T., Gross, M., Grosse-Wortmann, U., Grünert, J., Grunewald, S., Grzegory, P., Feng, G., Guler, H., Gusev, G., Gutierrez, J. L., Hagge, L., Hamberg, M., Hanneken, R., Harms, E., Hartl, I., Hauberg, A., Hauf, S., Hauschildt, J., Hauser, J., Havlicek, J., Hedqvist, A., Heidbrook, N., Hellberg, F., Henning, D., Hensler, O., Hermann, T., Hidvégi, A., Hierholzer, M., Hintz, H., Hoffmann, F., Hoffmann, M., Hoffmann, M., Holler, Y., Hüning, M., Ignatenko, A., Ilchen, M., Iluk, A., Iversen, J., Iversen, J., Izquierdo, M., Jachmann, L., Jardon, N., Jastrow, U., Jensch, K., Jensen, J., Jeżabek, M., Jidda, M., Jin, H., Johansson, N., Jonas, R., Kaabi, W., Kaefer, D., Kammering, R., Kapitza, H., Karabekyan, S., Karstensen, S., Kasprzak, K., Katalev, V., Keese, D., Keil, B., Kholopov, M., Killenberger, M., Kitaev, B., Klimchenko, Y., Klos, R., Knebel, L., Koch, A., Koepke, M., Köhler, S., Köhler, W., Kohlstrunk, N., Konopkova, Z., Konstantinov, A., Kook, W., Koprek, W., Körfer, M., Korth, O., Kosarev, A., Kosiński, K., Kostin, D., Kot, Y., Kotarba, A., Kozak, T., Kozak, V., Kramert, R., Krasilnikov, M., Krasnov, A., Krause, B., Kravchuk, L., Krebs, O., Kretschmer, R., Kreutzkamp, J., Kröplin, O., Krzysik, K., Kube, G., Kuehn, H., Kujala, N., Kulikov, V., Kuzminych, V., La Civita, D., Lacroix, M., Lamb, T., Lancetov, A., Larsson, M., Le Pinvidic, D., Lederer, S., Lensch, T., Lenz, D., Leuschner, A., Levenhagen, F., Li, Y., Liebing, J., Lilje, L., Limberg, T., Lipka, D., List, B., Liu, J., Liu, S., Lorbeer, B., Lorkiewicz, J., Lu, H. H., Ludwig, F., Machau, K., Maciocha, W., Madec, C., Magueur, C., Maiano, C., Maksimova, I., Malcher, K., Maltezopoulos, T., Mamoshkina, E., Manschwetus, B., Marcellini, F., Marinkovic, G., Martinez, T., Martirosyan, H., Maschmann, W., Maslov, M., Matheisen, A., Mavric, U., Meißner, J., Meissner, K., Messerschmidt, M., Meyners, N., Michalski, G., Michelato, P., Mildner, N., Moe, M., Moglia, F., Mohr, C., Mohr, S., Möller, W., Mommerz, M., Monaco, L., Montiel, C., Moretti, M., Morozov, I., Morozov, P., Mross, D., Mueller, J., Müller, C., Müller, J., Müller, K., Munilla, J., Münnich, A., Muratov, V., Napoly, O., Näser, B., Nefedov, N., Neumann, R., Neumann, R., Ngada, N., Noelle, D., Obier, F., Okunev, I., Oliver, J. A., Omet, M., Oppelt, A., Ottmar, A., Oublaid, M., Pagani, C., Paparella, R., Paramonov, V., Peitzmann, C., Penning, J., Perus, A., Peters, F., Petersen, B., Petrov, A., Petrov, I., Pfeiffer, S., Pflüger, J., Philipp, S., Pienaud, Y., Pierini, P., Pivovarov, S., Planas, M., Pławski, E., Pohl, M., Polinski, J., Popov, V., Prat, S., Prenting, J., Priebe, G., Pryschelski, H., Przygoda, K., Pyata, E., Racky, B., Rathjen, A., Ratuschni, W., Regnaud-Campderros, S., Rehlich, K., Reschke, D., Robson, C., Roever, J., Roggli, M., Rothenburg, J., Rusiński, E., Rybaniec, R., Sahling, H., Salmani, M., Samoylova, L., Sanzone, D., Saretzki, F., Sawlanski, O., Schaffran, J., Schlarb, H., Schlösser, M., Schlott, V., Schmidt, C., Schmidt-Foehre, F., Schmitz, M., Schmökel, M., Schnautz, T., Schneidmiller, E., Scholz, M., Schöneburg, B., Schultze, J., Schulz, C., Schwarz, A., Sekutowicz, J., Sellmann, D., Semenov, E., Serkez, S., Sertore, D., Shehzad, N., Shemarykin, P., Shi, L., Sienkiewicz, M., Sikora, D., Sikorski, M., Silenzi, A., Simon, C., Singer, W., Singer, X., Sinn, H., Sinram, K., Skvorodnev, N., Smirnow, P., Sommer, T., Sorokin, A., Stadler, M., Steckel, M., Steffen, B., Steinhau-Kühl, N., Stephan, F., Stodulski, M., Stolper, M., Sulimov, A., Susen, R., Świerblewski, J., Sydlo, C., Syresin, E., Sytchev, V., Szuba, J., Tesch, N., Thie, J., Thiebault, A., Tiedtke, K., Tischhauser, D., Tolkiehn, J., Tomin, S., Tonisch, F., Toral, F., Torbin, I., Trapp, A., Treyer, D., Trowitzsch, G., Trublet, T., Tschentscher, T., Ullrich, F., Vannoni, M., Varela, P., Varghese, G., Vashchenko, G., Vasic, M., Vazquez-Velez, C., Verguet, A., Vilcins-Czvitkovits, S., Villanueva, R., Visentin, B., Viti, M., Vogel, E., Volobuev, E., Wagner, R., Walker, N., Wamsat, T., Weddig, H., Weichert, G., Weise, H., Wenndorf, R., Werner, M., Wichmann, R., Wiebers, C., Wiencek, M., Wilksen, T., Will, I., Winkelmann, L., Winkowski, M., Wittenburg, K., Witzig, A., Wlk, P., Wohlenberg, T., Wojciechowski, M., Wolff-Fabris, F., Wrochna, G., Wrona, K., Yakopov, M., Yang, B., Yang, F., Yurkov, M., Zagorodnov, I., Zalden, P., Zavadtsev, A., Zavadtsev, D., Zhirnov, A., Zhukov, A., Ziemann, V., Zolotov, A., Zolotukhina, N., Zummack, F. & Zybin, D. (2020). *Nat. Photon.* **14**, 391–397.

[bb19] Dempster, A. P., Laird, N. M. & Rubin, D. B. (1977). *J. R. Stat. Soc. Ser. B*, **39**, 1–22.

[bb20] Egelman, E. H. (2016). *Biophys. J.* **110**, 1008–1012. 10.1016/j.bpj.2016.02.001 PMC4788751 26958874

[bb21] Ekeberg, T., Svenda, M., Abergel, C., Maia, F. R. N. C., Seltzer, V., Claverie, J.-M., Hantke, M., Jönsson, O., Nettelblad, C., van der Schot, G., Liang, M., DePonte, D. P., Barty, A., Seibert, M. M., Iwan, B., Andersson, I., Loh, N. D., Martin, A. V., Chapman, H., Bostedt, C., Bozek, J. D., Ferguson, K. R., Krzywinski, J., Epp, S. W., Rolles, D., Rudenko, A., Hartmann, R., Kimmel, N. & Hajdu, J. (2015). *Phys. Rev. Lett.* **114**, 098102. 10.1103/PhysRevLett.114.098102 25793853

[bb22] Ekeberg, T., Assalauova, D., Bielecki, J., Boll, R., Daurer, B. J., Eichacker, L. A., Franken, L. E., Galli, D. E., Gelisio, L., Gumprecht, L., Gunna, L. H., Hajdu, J., Hartmann, R., Hasse, D., Ignatenko, A., Koliyadu, J., Kulyk, O., Kurta, R., Kuster, M., Lugmayr, W., Lübkeh, J., Mancuso, A. P., Mazza, T., Nettelblad, C., Ovcharenko, Y., Rivas, D. E., Samanta, A. K., Schmidt, P., Sobolev, E., Timneanu, N., Usenko, S., Westphal, D., Wollweber, T., Worbs, L., Xavier, P. L., Yousef, H., Ayyer, K., Chapman, H. N., Sellberg, J. A., Seuring, C., Vartanyants, I. A., Küpper, J., Meyer, M. & Maia, F. R. N. C. (2022). *bioRxiv*, 2022.03.09.483477.

[bb23] Emma, P., Akre, R., Arthur, J., Bionta, R., Bostedt, C., Bozek, J., Brachmann, A., Bucksbaum, P., Coffee, R., Decker, F.-J., Ding, Y., Dowell, D., Edstrom, S., Fisher, A., Frisch, J., Gilevich, S., Hastings, J., Hays, G., Hering, P., Huang, Z., Iverson, R., Loos, H., Messerschmidt, M., Miahnahri, A., Moeller, S., Nuhn, H.-D., Pile, G., Ratner, D., Rzepiela, J., Schultz, D., Smith, T., Stefan, P., Tompkins, H., Turner, J., Welch, J., White, W., Wu, J., Yocky, G. & Galayda, J. (2010). *Nat. Photon.* **4**, 641–647.

[bb24] Fienup, J. R. (1982). *Appl. Opt.* **21**, 2758–2769. 10.1364/AO.21.002758 20396114

[bb25] Fienup, J. R. (2013). *Appl. Opt.* **52**, 45–56. 10.1364/AO.52.000045 23292374

[bb26] Füzik, T., Formanová, P., Růžek, D., Yoshii, K., Niedrig, M. & Plevka, P. (2018). *Nat. Commun.* **9**, 436. 10.1038/s41467-018-02882-0 PMC5789857 29382836

[bb27] Gaffney, K. J. & Chapman, H. N. (2007). *Science*, **316**, 1444–1448. 10.1126/science.1135923 17556577

[bb28] Giewekemeyer, K., Aquila, A., Loh, N.-T. D., Chushkin, Y., Shanks, K. S., Weiss, J. T., Tate, M. W., Philipp, H. T., Stern, S., Vagovic, P., Mehrjoo, M., Teo, C., Barthelmess, M., Zontone, F., Chang, C., Tiberio, R. C., Sakdinawat, A., Williams, G. J., Gruner, S. M. & Mancuso, A. P. (2019). *IUCrJ*, **6**, 357–365. 10.1107/S2052252519002781 PMC6503918 31098017

[bb29] Gorobtsov, O. Y., Lorenz, U., Kabachnik, N. M. & Vartanyants, I. A. (2015). *Phys. Rev. E*, **91**, 062712. 10.1103/PhysRevE.91.062712 26172741

[bb30] Gutt, C., Wochner, P., Fischer, B., Conrad, H., Castro-Colin, M., Lee, S., Lehmkühler, F., Steinke, I., Sprung, M., Roseker, W., Zhu, D., Lemke, H., Bogle, S., Fuoss, P. H., Stephenson, G. B., Cammarata, M., Fritz, D. M., Robert, A. & Grübel, G. (2012). *Phys. Rev. Lett.* **108**, 024801. 10.1103/PhysRevLett.108.024801 22324689

[bb31] Ignatenko, A., Assalauova, D., Bobkov, S. A., Gelisio, L., Teslyuk, A. B., Ilyin, V. A. & Vartanyants, I. A. (2021). *Mach. Learn. Sci. Technol.* **2**, 025014.

[bb32] Ishikawa, T., Aoyagi, H., Asaka, T., Asano, Y., Azumi, N., Bizen, T., Ego, H., Fukami, K., Fukui, T., Furukawa, Y., Goto, S., Hanaki, H., Hara, T., Hasegawa, T., Hatsui, T., Higashiya, A., Hirono, T., Hosoda, N., Ishii, M., Inagaki, T., Inubushi, Y., Itoga, T., Joti, Y., Kago, M., Kameshima, T., Kimura, H., Kirihara, Y., Kiyomichi, A., Kobayashi, T., Kondo, C., Kudo, T., Maesaka, H., Maréchal, X. M., Masuda, T., Matsubara, S., Matsumoto, T., Matsushita, T., Matsui, S., Nagasono, M., Nariyama, N., Ohashi, H., Ohata, T., Ohshima, T., Ono, S., Otake, Y., Saji, C., Sakurai, T., Sato, T., Sawada, K., Seike, T., Shirasawa, K., Sugimoto, T., Suzuki, S., Takahashi, S., Takebe, H., Takeshita, K., Tamasaku, K., Tanaka, H., Tanaka, R., Tanaka, T., Togashi, T., Togawa, K., Tokuhisa, A., Tomizawa, H., Tono, K., Wu, S., Yabashi, M., Yamaga, M., Yamashita, A., Yanagida, K., Zhang, C., Shintake, T., Kitamura, H. & Kumagai, N. (2012). *Nat. Photon.* **6**, 540–544.

[bb33] Kang, H.-S., Min, C.-K., Heo, H., Kim, C., Yang, H., Kim, G., Nam, I., Baek, S. Y., Choi, H.-J., Mun, G., Park, B. R., Suh, Y. J., Shin, D. C., Hu, J., Hong, J., Jung, S., Kim, S.-H., Kim, K., Na, D., Park, S. S., Park, Y. J., Han, J.-H., Jung, Y. G., Jeong, S. H., Lee, H. G., Lee, S., Lee, S., Lee, W.-W., Oh, B., Suh, H. S., Parc, Y. W., Park, S.-J., Kim, M. H., Jung, N.-S., Kim, Y.-C., Lee, M.-S., Lee, B.-H., Sung, C.-W., Mok, I.-S., Yang, J.-M., Lee, C.-S., Shin, H., Kim, J. H., Kim, Y., Lee, J. H., Park, S.-Y., Kim, J., Park, J., Eom, I., Rah, S., Kim, S., Nam, K. H., Park, J., Park, J., Kim, S., Kwon, S., Park, S. H., Kim, K. S., Hyun, H., Kim, S. N., Kim, S., Hwang, S., Kim, M. J., Lim, C., Yu, C.-J., Kim, B.-S., Kang, T.-H., Kim, K.-W., Kim, S.-H., Lee, H.-S., Lee, H.-S., Park, K.-H., Koo, T.-Y., Kim, D.-E. & Ko, I. S. (2017). *Nat. Photon.* **11**, 708–713.

[bb35] Kurta, R. P., Donatelli, J. J., Yoon, C. H., Berntsen, P., Bielecki, J., Daurer, B. J., DeMirci, H., Fromme, P., Hantke, M. F., Maia, F. R. N. C., Munke, A., Nettelblad, C., Pande, K., Reddy, H. K. N., Sellberg, J. A., Sierra, R. G., Svenda, M., van der Schot, G., Vartanyants, I. A., Williams, G. J., Xavier, P. L., Aquila, A., Zwart, P. H. & Mancuso, A. P. (2017). *Phys. Rev. Lett.* **119**, 158102. 10.1103/PhysRevLett.119.158102 PMC5757528 29077445

[bb36] Loh, N. D. & Elser, V. (2009). *Phys. Rev. E*, **80**, 026705. 10.1103/PhysRevE.80.026705 19792279

[bb37] Long, F., Doyle, M., Fernandez, E., Miller, A. S., Klose, T., Sevvana, M., Bryan, A., Davidson, E., Doranz, B. J., Kuhn, R. J., Diamond, M. S., Crowe, J. E. & Rossmann, M. G. (2019). *Proc. Natl Acad. Sci. USA*, **116**, 1591–1596. 10.1073/pnas.1815432116 PMC6358714 30642974

[bb38] Lundholm, I. V., Sellberg, J. A., Ekeberg, T., Hantke, M. F., Okamoto, K., van der Schot, G., Andreasson, J., Barty, A., Bielecki, J., Bruza, P., Bucher, M., Carron, S., Daurer, B. J., Ferguson, K., Hasse, D., Krzywinski, J., Larsson, D. S. D., Morgan, A., Mühlig, K., Müller, M., Nettelblad, C., Pietrini, A., Reddy, H. K. N., Rupp, D., Sauppe, M., Seibert, M., Svenda, M., Swiggers, M., Timneanu, N., Ulmer, A., Westphal, D., Williams, G., Zani, A., Faigel, G., Chapman, H. N., Möller, T., Bostedt, C., Hajdu, J., Gorkhover, T. & Maia, F. R. N. C. (2018). *IUCrJ*, **5**, 531–541. 10.1107/S2052252518010047 PMC6126651 30224956

[bb39] Lyumkis, D. (2019). *J. Biol. Chem.* **294**, 5181–5197. 10.1074/jbc.REV118.005602 PMC6442032 30804214

[bb40] Mancuso, A. P., Aquila, A., Batchelor, L., Bean, R. J., Bielecki, J., Borchers, G., Doerner, K., Giewekemeyer, K., Graceffa, R., Kelsey, O. D., Kim, Y., Kirkwood, H. J., Legrand, A., Letrun, R., Manning, B., Lopez Morillo, L., Messerschmidt, M., Mills, G., Raabe, S., Reimers, N., Round, A., Sato, T., Schulz, J., Signe Takem, C., Sikorski, M., Stern, S., Thute, P., Vagovič, P., Weinhausen, B. & Tschentscher, T. (2019). *J. Synchrotron Rad.* **26**, 660–676. 10.1107/S1600577519003308 PMC6510195 31074429

[bb41] Marchesini, S., He, H., Chapman, H. N., Hau-Riege, S. P., Noy, A., Howells, M. R., Weierstall, U. & Spence, J. C. H. (2003). *Phys. Rev. B*, **68**, 140101.

[bb42] Merkel, D. (2014). *Linux J.* **2014**, 2.

[bb43] Miao, J., Charalambous, P., Kirz, J. & Sayre, D. (1999). *Nature*, **400**, 342–344.

[bb44] Miao, J., Ishikawa, T., Robinson, I. K. & Murnane, M. M. (2015). *Science*, **348**, 530–535. 10.1126/science.aaa1394 25931551

[bb45] Neira, J. L. (2013). *Structure and Physics of Viruses – an Integrated Textbook*, edited by M. J. Mateu, pp. 177–202. Madrid: Springer.

[bb46] Neutze, R., Wouts, R., van der Spoel, D., Weckert, E. & Hajdu, J. (2000). *Nature*, **406**, 752–757. 10.1038/35021099 10963603

[bb47] Pichkur, E. B., Samygina, V. R., Ivanova, A. L., Fedotov, A. Y., Ivanov, A. P., Khvatov, E. V., Ishmukhametov, A. A. & Vorovich, M. F. (2020). *Crystallogr. Rep.* **65**, 915–921.

[bb48] Pierson, T. C. & Diamond, M. S. (2020). *Nat. Microbiol.* **5**, 796–812. 10.1038/s41564-020-0714-0 PMC7696730 32367055

[bb49] Poudyal, I., Schmidt, M. & Schwander, P. (2020). *Struct. Dyn.* **7**, 024102. 10.1063/1.5144516 PMC7088463 32232074

[bb50] Rey, F. A., Stiasny, K., Vaney, M., Dellarole, M. & Heinz, F. X. (2018). *EMBO Rep.* **19**, 206–224. 10.15252/embr.201745302 PMC5797954 29282215

[bb51] Rose, M., Bobkov, S., Ayyer, K., Kurta, R. P., Dzhigaev, D., Kim, Y. Y., Morgan, A. J., Yoon, C. H., Westphal, D., Bielecki, J., Sellberg, J. A., Williams, G., Maia, F. R. N. C., Yefanov, O. M., Ilyin, V., Mancuso, A. P., Chapman, H. N., Hogue, B. G., Aquila, A., Barty, A. & Vartanyants, I. A. (2018). *IUCrJ*, **5**, 727–736. 10.1107/S205225251801120X PMC6211532 30443357

[bb52] Rossmann, M. G. (2014). *Phys. Scr.* **89**, 098005.

[bb53] Seibert, M. M., Ekeberg, T., Maia, F. R. N. C., Svenda, M., Andreasson, J., Jönsson, O., Odić, D., Iwan, B., Rocker, A., Westphal, D., Hantke, M., DePonte, D. P., Barty, A., Schulz, J., Gumprecht, L., Coppola, N., Aquila, A., Liang, M., White, T. A., Martin, A., Caleman, C., Stern, S., Abergel, C., Seltzer, V., Claverie, J.-M., Bostedt, C., Bozek, J. D., Boutet, S., Miahnahri, A. A., Messerschmidt, M., Krzywinski, J., Williams, G., Hodgson, K. O., Bogan, M. J., Hampton, C. Y., Sierra, R. G., Starodub, D., Andersson, I., Bajt, S., Barthelmess, M., Spence, J. C. H., Fromme, P., Weierstall, U., Kirian, R., Hunter, M., Doak, R. B., Marchesini, S., Hau-Riege, S. P., Frank, M., Shoeman, R. L., Lomb, L., Epp, S. W., Hartmann, R., Rolles, D., Rudenko, A., Schmidt, C., Foucar, L., Kimmel, N., Holl, P., Rudek, B., Erk, B., Hömke, A., Reich, C., Pietschner, D., Weidenspointner, G., Strüder, L., Hauser, G., Gorke, H., Ullrich, J., Schlichting, I., Herrmann, S., Schaller, G., Schopper, F., Soltau, H., Kühnel, K.-U., Andritschke, R., Schröter, C.-D., Krasniqi, F., Bott, M., Schorb, S., Rupp, D., Adolph, M., Gorkhover, T., Hirsemann, H., Potdevin, G., Graafsma, H., Nilsson, B., Chapman, H. N. & Hajdu, J. (2011). *Nature*, **470**, 78–81.

[bb54] Shi, Y., Yin, K., Tai, X., DeMirci, H., Hosseinizadeh, A., Hogue, B. G., Li, H., Ourmazd, A., Schwander, P., Vartanyants, I. A., Yoon, C. H., Aquila, A. & Liu, H. (2019). *IUCrJ*, **6**, 331–340. 10.1107/S2052252519001854 PMC6400180 30867930

[bb55] Singer, A., Sorgenfrei, F., Mancuso, A. P., Gerasimova, N., Yefanov, O. M., Gulden, J., Gorniak, T., Senkbeil, T., Sakdinawat, A., Liu, Y., Attwood, D., Dziarzhytski, S., Mai, D. D., Treusch, R., Weckert, E., Salditt, T., Rosenhahn, A., Wurth, W. & Vartanyants, I. A. (2012). *Opt. Express*, **20**, 17480. 10.1364/OE.20.017480 23038301

[bb56] Sobolev, E., Zolotarev, S., Giewekemeyer, K., Bielecki, J., Okamoto, K., Reddy, H. K. N., Andreasson, J., Ayyer, K., Barak, I., Bari, S., Barty, A., Bean, R., Bobkov, S., Chapman, H. N., Chojnowski, G., Daurer, B. J., Dörner, K., Ekeberg, T., Flückiger, L., Galzitskaya, O., Gelisio, L., Hauf, S., Hogue, B. G., Horke, D. A., Hosseinizadeh, A., Ilyin, V., Jung, C., Kim, C., Kim, Y., Kirian, R. A., Kirkwood, H., Kulyk, O., Küpper, J., Letrun, R., Loh, N. D., Lorenzen, K., Messerschmidt, M., Mühlig, K., Ourmazd, A., Raab, N., Rode, A. V., Rose, M., Round, A., Sato, T., Schubert, R., Schwander, P., Sellberg, J. A., Sikorski, M., Silenzi, A., Song, C., Spence, J. C. H., Stern, S., Sztuk-Dambietz, J., Teslyuk, A., Timneanu, N., Trebbin, M., Uetrecht, C., Weinhausen, B., Williams, G. J., Xavier, P. L., Xu, C., Vartanyants, I. A., Lamzin, V. S., Mancuso, A. & Maia, F. R. N. C. (2020). *Commun. Phys.* **3**, 97.

[bb57] Vartanyants, I. A., Singer, A., Mancuso, A. P., Yefanov, O. M., Sakdinawat, A., Liu, Y., Bang, E., Williams, G. J., Cadenazzi, G., Abbey, B., Sinn, H., Attwood, D., Nugent, K. A., Weckert, E., Wang, T., Zhu, D., Wu, B., Graves, C., Scherz, A., Turner, J. J., Schlotter, W. F., Messerschmidt, M., Lüning, J., Acremann, Y., Heimann, P., Mancini, D. C., Joshi, V., Krzywinski, J., Soufli, R., Fernandez-Perea, M., Hau-Riege, S., Peele, A. G., Feng, Y., Krupin, O., Moeller, S. & Wurth, W. (2011). *Phys. Rev. Lett.* **107**, 144801. 10.1103/PhysRevLett.107.144801 22107200

[bb58] Yang, H. & Rao, Z. (2021). *Nat. Rev. Microbiol.* **19**, 685–700. 10.1038/s41579-021-00630-8 PMC8447893 34535791

[bb59] Zhang, X., Jia, R., Shen, H., Wang, M., Yin, Z. & Cheng, A. (2017). *Viruses*, **9**, 338. 10.3390/v9110338 PMC5707545 29137162

